# SREBF1-mediated SND1 transcriptional activation promotes prostate cancer progression via MTDH interaction through the SESN2/AMPK/mTOR axis

**DOI:** 10.1186/s12967-025-06762-2

**Published:** 2025-08-07

**Authors:** Yijie Tang, Yufan Ying, Xueyou Ma, Jiahe Yi, Zixiang Liu, Yuqing Wu, Shen Lin, Xuan Shu, Zhixiang Qi, Jindan Luo, Xiangyi Zheng, Jiangfeng Li, Liping Xie, Ben Liu

**Affiliations:** 1 Department of Urology, First Affiliated Hospital, School of Medicine, Zhejiang University, Hangzhou, Zhejiang 310000 China; 2 Cancer Center, Zhejiang University, Hangzhou, Zhejiang 310058 China

**Keywords:** Prostate cancer, SND1, MTDH, SESN2, mTOR

## Abstract

**Background:**

Prostate cancer (PCa) is a prevalent cancer and a major cause of cancer-related deaths in men worldwide. Growing evidence indicates that Staphylococcal nuclease and Tudor domain containing 1 (SND1) is a multifunctional protein extensively involved in transcriptional regulation, RNA maturation, post-transcriptional modifications, and other processes. However, previous studies have rarely investigated the function of SND1 as an RNA-binding protein in PCa tumorigenesis.

**Methods:**

The Cancer Genome Atlas and NCBI Gene Expression Omnibus (GEO) databases were used to evaluate SND1 expression levels in PCa. We conducted a series of in vitro and in vivo functional experiments to assess the biological functions of SND1, including cell counting kit-8, colony formation, Transwell and wound-healing assays, and animal experiments in nude mice. Chromatin immunoprecipitation, dual-luciferase reporter assay, and DNA pull-down assay were performed to validate the association between the upstream transcription factor and SND1. Based on mass spectrometry, RNA-seq, and RNA immunoprecipitation **(**RIP)-seq, we identified the downstream targets of SND1- Sestrin 2 (SESN2), which were validated through qRT-PCR, Western blotting, RIP-qPCR, dual-luciferase reporter assay, and RNA pull-down assay. Finally, a series of functional assays and Western blotting analyses confirmed SESN2 as a downstream target of SND1.

**Results:**

Our research identified that SND1 was significantly elevated in PCa, and knocking down SND1 repressed PCa multiplication and migration. Mechanistically, sterol regulatory element binding transcription factor 1 (SREBF1) bound to the promoter of the SND1 gene and activated its transcription, which subsequently formed a complex with metadherin (MTDH). This complex is directly bound to and degraded SESN2 mRNA, and disruption of this interaction with C26-A6 inhibited MTDH-SND1-mediated SESN2 degradation. Notably, SESN2 expression was inhibited in PCa and may exert tumor-suppressive effects by affecting the AMPK/mTOR signaling pathway. Rescue experiments indicated that knocking down SND1 or MTDH significantly inhibited PCa proliferation and migration, and knocking down SESN2 partially reversed this effect.

**Conclusions:**

Our study reveals SND1 overexpression in PCa, which is transcriptionally activated by SREBF1. Mechanistically, SND1 interacts with MTDH and promotes SESN2 mRNA degradation, modulating PCa progression through the AMPK/mTOR pathway.

**Supplementary Information:**

The online version contains supplementary material available at 10.1186/s12967-025-06762-2.

## Graphical abstract

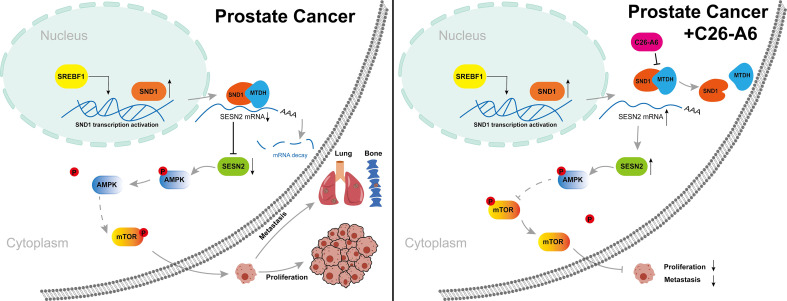

## Introduction

To date, the incidence of prostate cancer (PCa) among male malignancies is ranked first globally [1]. With economic development and improving living conditions, its incidence in China has been rapidly increasing in recent years [2]. Nevertheless, there are still many challenges in the pathogenesis of PCa and therapies targeting castration-resistant prostate cancer (CRPC) [3]. Therefore, it is imperative to further explore this field and discover more drug targets.

A multitude of research has documented that Staphylococcal nuclease and Tudor domain containing 1 (SND1), a protein with evolutionary conservation, plays a significant role in transcription regulation [4], alternative splicing [5, 6], microRNA decay [7], formation of RNA-induced silencing complex (RISC) [8], RNA stabilization [9, 10], and stress response [11, 12]. Regarding the role of SND1 in tumors, it has been reported that SND1 promotes tumor growth by activating Wnt signaling [13]. Yu et al. reported that the TGFβ1/SND1/Smurf1 axis facilitates breast cancer metastasis [14]. Yoo et al. reported that SND1 and metadherin (MTDH) form a complex that enhances the degradation of tumor suppressor mRNAs [15]. It has also been reported that C26-A6 and C26-A2 inhibit tumor growth and metastasis by targeting and disrupting the MTDH-SND1 complex [16]. Zheng et al. reported that SND1 promotes glioma progression by recognizing the m^6^A site of Nrf2 mRNA [17]; Baquero-Perez et al. demonstrated that SND1 acts as an m^6^A modification reader to facilitate the replication of Kaposi’s sarcoma-associated herpesviru*s* [18]. Our previous studies have explored the mechanisms of m^6^A readers such as YTHDF*2* and IGF2BPs in urinary system tumors [19, 20, 21]. Nevertheless, the mechanism by which SND1, a well-known RNA-binding protein (RBP), promotes PCa progression remains unclear.

Sestrin 2 (SESN2) is an essential protein involved in the cellular stress response, and it holds great importance in adaptation to environmental stresses, including oxidation, nutrient deprivation, DNA damage, and others [22, 23]. Liang et al. reported that isorhapontigenin-induced SESN2 expression exerted an inhibitory effect on human bladder cancer [24]. Li et al. revealed that SESN2 activates AMPK and inhibits mTOR, inducing autophagy and sustaining insulin sensitivity [25]. Besides, Bodmer et al. reported that the balance between survival and apoptosis in sensory hair cells during stress is regulated by the Sesn2/AMPK/mTOR signaling axis [26]. Moreover, aberrant mTOR signaling has been observed in various tumors, where its activation promotes tumor growth and metastasis [27, 28, 29]. According to the studies mentioned, SESN2 functions as a tumor suppressor gene in specific cancers, possibly through the AMPK/mTOR signaling pathway.

Our study found that SND1 was upregulated in PCa. Functionally, the knockdown (KD) of SND1 suppressed the proliferation and migration of PCa cells. Mechanistically, SND1 was activated by SREBF1, which then interacted with MTDH to promote the decay of SESN2 mRNA, inhibit AMPK phosphorylation, and activate the mTOR signaling pathway, thereby promoting PCa progression. Collectively, we found that the SREBF1/SND1/SESN2/APMK/mTOR axis was responsible for the malignancy of PCa, which may present new strategies for treating PCa.

## Materials and methods

### Clinical samples

Clinical samples used in our study were obtained from 32 patients with PCa at the First Affiliated Hospital of Medical College, Zhejiang University (Hangzhou, China). Informed consent was obtained from each participant in writing. The entire process of our research was conducted in accordance with the Declaration of Helsinki. The study was conducted in full compliance with ethical guidelines and received ethical approval.

### Cell lines

22RV1, DU-145, PC-3, and RWPE-1 cell lines were procured from the National Collection of Authenticated Cell Cultures (Shanghai, China) and cultured at 37 ℃ in a CO_2_ incubator following the manufacturer’s guidelines, aligning with the methodologies employed in our prior research [20].

### Reagents and transfection

The lentivirus-based short hairpin RNA (shRNA) targeting SND1 and MTDH, along with overexpression plasmids, were all bought from GeneCopoeia (Guangzhou, China). The small interfering RNAs (siRNAs), specifically designed to target the genes SND1, MTDH, and SESN2, were sourced commercially through RIBOBIO (Guangzhou, China). Transfection was executed using the jetPRIME Polyplus reagent kit (France), strictly following the manufacturer’s standard operating procedure. The sequences of the siRNAs used and the core sequences of the shRNAs are detailed in Supplementary Table 1.

### RNA isolation and qRT-PCR

The experimental procedures and data analysis methods were adapted from our previous study. The specific procedures for qRT-PCR were as follows: Cells were cultured in 6-well plates and lysed using RNA isolater Total RNA Extraction Reagent (R401-01, Vazyme). RNA extraction was performed strictly according to the TRIzol method. After drying, the RNA was dissolved in DEPC-treated water, and the concentration was measured. Reverse transcription was performed using HiScript II Q RT SuperMix for quantitative real-time PCR (qPCR) (R223-01, Vazyme). qPCR was conducted using ChamQ Universal SYBR qPCR Master Mix (R711-02, Vazyme) on a CFX96 Touch Real-Time PCR Detection System (Bio-Rad). β-Actin was selected as the endogenous reference gene, and the relative expression levels of target genes were calculated using the 2^− ΔΔ𝐶𝑡^ method. The primer sequences used are presented in Supplementary Table 1.

### Western blotting

The procedures were meticulously followed, as outlined in our previous study [19]. The specific procedures were as follows: Cells were cultured in 6-well plates and lysed using RIPA buffer containing protease (FD1001, FUDE) and phosphatase (FD1001, FUDE) inhibitors. The total protein concentrationwas determined using a BCA protein assay kit (FD2001, FUDE). Subsequently, a 1/4 volume of 5× loading buffer was added, mixed thoroughly, centrifuged, andheated at 100 ℃ for 5 min to denature the proteins. Next, samples were loaded onto a pre-cast 10% SDS-PAGE gel (FD341, FUDE) and subjected to electrophoresis at 200 V for 45 min, then electro-transferred onto a PVDF membrane (#IPVH00010, Merck Millipore) at 300 mA for 1 h. After that, PVDF membranes were blocked with skim milk powder, dissolved in TBST (FD0080, FUDE) for 1 h at room temperature, and incubated with primary antibodies at 4 ℃ overnight. Finally, the membrane was washed using TBST for 30 min, incubated with HRP-conjugated secondary antibodies (FDR007, FDM007) for 1 h at room temperature, and washed using TBST for 30 min again. The protein bands were visualized using a Bio-Rad ChemiDoc MP imaging system (Bio-Rad). Detailed information on the antibodies (including the manufacturer, catalog number, purpose, and dilution ratio) is presented in Supplementary Table 1.

### Cell counting kit (CCK)-8, colony formation, transwell, and wound-healing assays

The CCK-8 assay was performed using the CCK-8 kit (FD3788-1000T, FUDE), and all procedures were conducted according to standard protocols [19]. All experiments were performed with at least three biological replicates. siNC (5’-UUCUCCGAACGUGUCACGU-3’) and empty pcDNA3.1 vector were used as negative controls.

### EdU assay

The experiment was conducted according to the protocol provided by the EdU kit (C0078S, Beyotime). Briefly, a certain number of cells were seeded into confocal dishes. After 48 h of treatment, the cells were incubated with 10 µM EdU solution for at least 2 h. The cells were then fixed with 4% paraformaldehyde and permeabilized with 0.3% Triton X-100. After three washes, the cells were stained with Hoechst 33,342 for 0.5 h. Finally, images were captured using a Leica laser confocal microscope. The experiment was performed with three biological replicates. siNC (5’-UUCUCCGAACGUGUCACGU-3’) and empty pcDNA3.1 vector were used as negative controls.

### Cell cycle analysis

Cells were transfected with either siNC or siRNAs and collected 48 h post-transfection. After centrifugation and three washes with PBS, the cells were fixed in 75% ethanol and stored at 4 ℃ for a minimum of 12 h. The next day, the cells were stained according to the manufacturer’s protocol (#CCS012, Multi Science) and analyzed using a BD LSR II Flow Cytometer System (BD Biosciences). The experiment was performed with three biological replicates. siNC (5’-UUCUCCGAACGUGUCACGU-3’) were used as negative controls.

### Animal experiments

Stable SND1 knockdown (shSND1) in PC-3 cells was achieved using shRNAs for subsequent animal studies. All animal experiments were conducted using 4-week-old male nude mice. For subcutaneous transplantation models, approximately 5 × 10^6^ cells were inoculated subcutaneously into each mouse using needles. Subsequently, tumor volume was assessed every four days using the following formula: V = length × width^2^ × 0.52. On the 40th day, all mice were sacrificed and dissected for subcutaneous tumors. Subsequently, the xenografts were anatomized, isolated, and weighed. Moreover, a pair of xenografts from shNC and shSND1 groups was used for IHC staining. For caudal vein tumorigenesis experiments, approximately 2 × 10^6^ PC-3-luci cells per plate were injected into the caudal veins. After about 40 days, the whole metastasis condition and every metastatic site were evaluated and imaged using the IVIS imaging system (PerkinElmer). After imaging, metastatic organs were anatomized for further imaging and sent for hematoxylin-eosin (H&E) staining. All procedures were strictly in accordance with animal ethical standards.

### Chromatin immunoprecipitation (ChIP)

Cells were seeded in 15 cm dishes and harvested at approximately 90% confluency. After fixation with 1% paraformaldehyde for approximately 20 min, the ChIP assay was performed using the SimpleChIP^®^ Enzymatic Chromatin IP Kit (9003, CST). The eluted ChIP-DNA was amplified with PCR for further analysis.

### RNA immunoprecipitation (RIP)

This experiment was performed strictly following the protocol of the RIP kit (17–701, Merck Millipore). Cells were first cultured in 15 cm plates and lysed at approximately 90% confluence. The magnetic beads were first incubated with 5 µg of antibodies targeting SND1, MTDH, and corresponding IgG for 0.5 h at 25 ℃. Subsequently, the cell lysates were centrifuged, and the supernatant was allowed to interact with the prepped magnetic beads for at least 10 h at 4 °C. The next day, the beads were digested with proteinase K after elution, and the enriched RNAs were isolated and purified for further sequencing or qRT-PCR.

### DNA pull-down assay

First, the sequence of SREBF1 binding to the SND1 promoter region was amplified by PCR and labeled with biotin (Cosmos Wisdom, Hangzhou, China). The biotin-labeled probe was then incubated with Streptavidin MagPoly Beads (SM007000, Smart-Lifesciences). Cells were lysed at 90% confluency and incubated with a biotin-labeled probe & Streptavidin MagPoly Beads complex. All remaining procedures were executed according to the manufacturer’s instructions of the Smart Streptavidin Magnetic IP/Co-IP Kit (SM017K01, Smart-Lifesciences).

### RNA pull-down assay

This experiment was performed strictly following the protocol (20164, Thermo Fisher Scientific). Briefly, 50 pmol biotinylated SESN2 RNA probes were incubated with cells overnight. Thereafter, the cells were lysed and incubated with 50 µL washed streptavidin beads for 1 h at 25 ℃. After three washes, the beads were boiled and subjected to Western blotting.

### Dual-luciferase reporter assay

The reporter plasmids carrying target sequences were synthesized by Tsingke (Beijing, China). Cells were plated into 24- or 96-well dishes and co-transfected with plasmids and siRNAs using the Polyplus transfection reagent. After transfection for 48 h, the activities of firefly and Renilla luciferases were detected using the Dual-Luciferase Reporter System (Promega, USA).

### Co-immunoprecipitation (Co-IP)

Co-IP was conducted following the guidelines provided (88828, Thermo Fisher Scientific). First, cells were seeded in 10–15 cm dishes and harvested at 80%–90% confluency. Second, the cells were lysed for 5 min, followed by centrifugation for 15 min. Meanwhile, a certain volume of beads was mixed with 2 µg of SND1 or MTDH antibody at 25 ℃ for 1 h. The protein supernatant and bead–antibody complex were then mixed and incubated overnight at 4 ℃. After three washes, the protein-antibody-bead complex was eluted with elution buffer, and bound proteins and 10% input were detected by LC/MS and confirmed by Western blotting.

### Immunofluorescence (IF)

Cells were seeded in cell culture dishes (Biosharp, BS-15-GJM) and incubated at 37 ℃. At 20%–30% confluency, the cells were removed from the incubator and washed thrice with PBS. Cells were then treated with 4% paraformaldehyde for fixation, 0.1% Triton X-100 for permeabilization, and 1% BSA for blocking. After incubation with the SND1 antibody, the cells were placed at 4 ℃ overnight. Similarly, after three washes with PBS, the cells were incubated with a fluorescent secondary antibody. After staining with DAPI, the cells were photographed using IF microscopy.

### Nuclear and cytoplasmic extract isolation

Cells were harvested at 90% confluence with curets. The experiment was conducted strictly following the protocols provided by the manufacturer (P0027, Beyotime).

### Online websites

Online tools used in our research were as follows: JASPAR (https://jaspar.elixir.no/), hTFtarget (https://guolab.wchscu.cn/hTFtarget/#!/), TFDB (https://guolab.wchscu.cn), GeneCards (https://www.genecagen.org/), and ENCORI (https://rnasysu.com/encori/).

### Statistical analysis

Data analysis was performed using GraphPad Prism software (version 8.0). The data are presented as mean ± standard deviation. A two-tailed unpaired t-test was used to assess the differences between the two groups. Statistical significance was defined as *P* < 0.05 (*P < 0.05, **P < 0.01, ***P < 0.001).

## Results

### SND1 was generally upregulated in cell lines and clinical samples

**Fig. 1:**
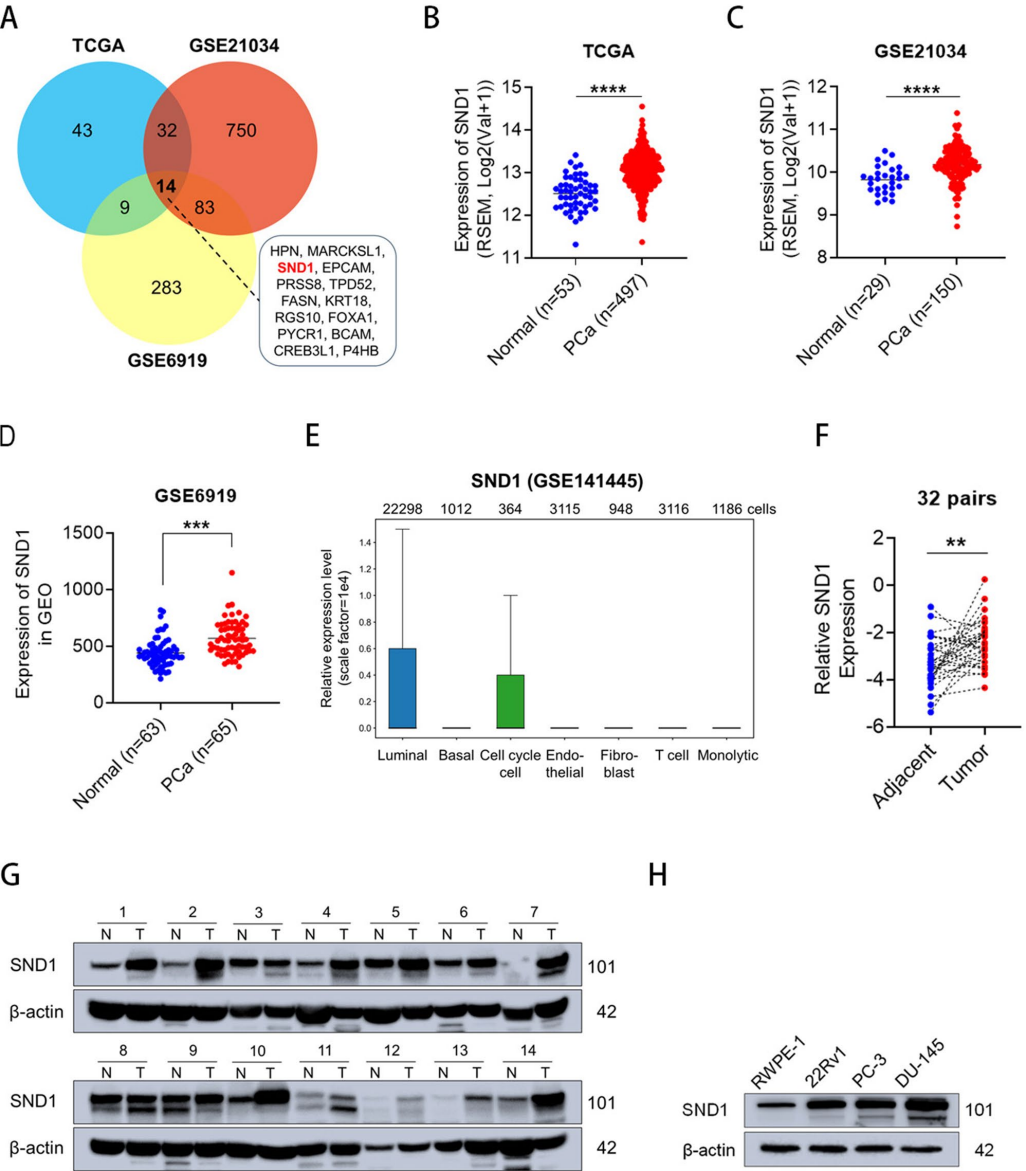
The expression of SND1 is elevated in PCa clinical samples and cell lines. **A** The Venn diagram shows the overlap of upregulated genes across TCGA, GSE21034, and GSE6919. **B** The expression mode of SND1 was analyzed between 497 PCa tissues and 53 adjacent normal prostate tissues (TCGA database). **C**, **D** The data from GSE21034 and GSE6919 revealed higher expression of SND1 in PCa tissues in comparison to benign tissues. **E** Single-cell sequencing data from GSE141445 showed that the relative expression level of SND1 in different types of PCa cells. **E** In our analysis of 32 clinical PCa samples, along with their paired normal tissues, SND1 levels were increased in 22 cases (68.75%). **G** Fourteen pairs of PCa samples were subject to western blot analysis of SND1. **H** Expression level of SND1 protein in PCa cell lines and RWPE-1 were analyzed. β-actin acted as internal reference

To identify abnormally expressed genes in PCa, data from the TCGA Prostate Adenocarcinoma Group, GSE21034, and GSE6919 datasets were downloaded and analyzed using Python 3.9. With screening criteria set as an adjusted *P* value < 0.01 and log_2 *(*_Fold Change) > 1.2, a total of 14 candidate differentially expressed genes were identified (Fig. 1A). Given that previous studies have demonstrated that SND1 functions as an RBP, we selected it as the target gene to investigate its role and mechanism as an RBP in PCa progression. To explore SND1 expression in PCa, we downloaded publicly available data from The Cancer Genome Atlas (TCGA), GSE21034 and GSE6919 databases, which exhibited that the SND1 expression was elevated in PCa tissues relative to normal prostate tissues (Fig. 1B-D). Single-cell sequencing data from GSE141445 (scCancerExplorer, https://bianlab.cn/scCancerExplorer/home) showed that the relative expression level of SND1 in luminal-type cells was significantly higher than that in other cell types, followed by Cell_cycle cells (Fig. 1E). The high expression of SND1 in luminal-type cells suggests that it may be closely associated with the biological behaviors of these cells. To further confirm the differential expression of SND1 between PCa and benign prostate tissues, we performed qRT-PCR and Western blotting based on 32 paired clinical PCa samples. These results were consistent with those obtained from TCGA and GEO datasets (Fig. 1F-G). Additionally, as revealed in Fig. 1H, SND1 was significantly upregulated in PCa cell lines compared to RWPE-1.

### SND1 KD inhibited PCa progression

**Fig. 2:**
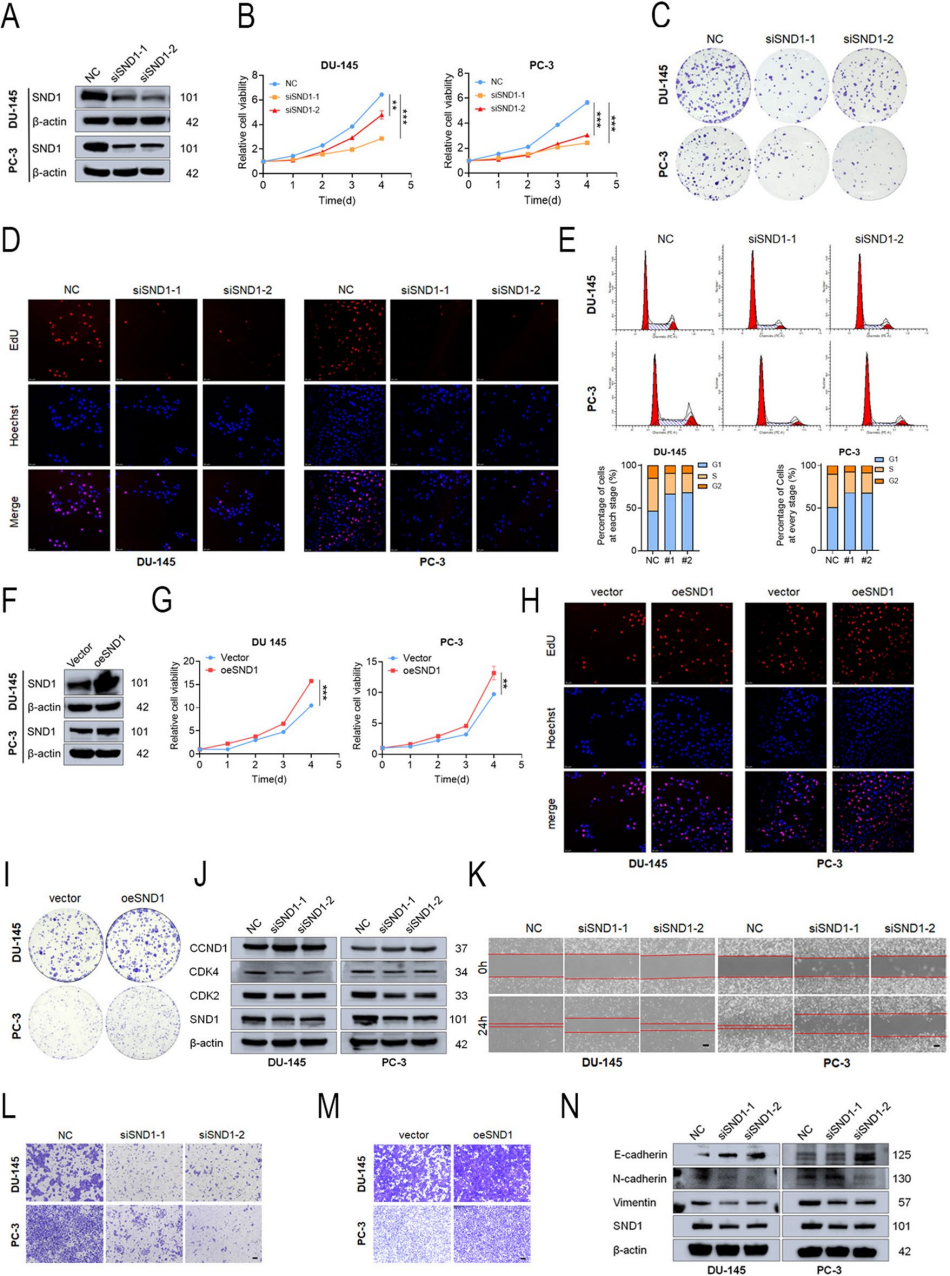
Knockdown of SND1 inhibits proliferation and migration of PCa cells in vitro. **A** Knockdown of SND1 was verified by western blot. **B**-**D** Knockdown of SND1 suppressed PCa proliferation evaluated by CCK-8, EdU and colony formation assay. **E** Cell cycle analysis indicated a notable increase of cells arrested in G1 phase in SND1 KD cells. **F** Overexpression of SND1 was verified by western blot. **G**-**I** Overexpression of SND1 promoted PCa proliferation and migration. **J** The expression of CDK2, CDK4, CCND1 was detected by western blot upon SND1 KD. **K**, **L** Knockdown of SND1 repressed PCa migration evaluated by trans-well assay and wound-healing assay (scale bar = 250 μm). **M** Overexpressing SND1 with plasmids promoted PCa migration evaluated by trans-well assay. **N** The expression of **E**-cadherin, N-cadherin and vimentin were detected by western blot upon SND1 KD. β-actin was the internal reference

Based on the high expression of SND1, we aimed to investigate its biological functions in DU-145 and PC-3 cells. Initially, siRNAs targeting SND1 and an overexpression plasmid were used to suppress or enhance SND1 expression in vitro. The efficiency of KD and overexpression was subsequently validated by Western blotting (Figs. 2A and F). Subsequently, CCK-8, colony formation, and EdU assays demonstrated significant inhibition of the proliferative capacity of PCa cells following SND1 KD (Figs. 2B–D and S2A-B). Cell cycle analysis revealed that SND1 KD resulted in cell cycle arrest at the G1 phase (Fig. 2E). Conversely, SND1 overexpression led to the opposite results, significantly enhancing the proliferative capacity of the cells (Figs. 2G–I and S2C-D). Consistent with these findings, Western blotting suggested that the expression levels of downstream CDK2 and CDK4 were significantly downregulated following SND1 KD (Fig. 2J). Contrarily, Transwell and wound-healing assays revealed the suppressed migratory capacity of PCa cells upon SND1 KD (Figs. 2K-L and S2E-F). Similarly, SND1 overexpression resulted in the opposite effect, significantly enhancing the migratory capacity of the cells (Figs. 2M and S2G). Based on SND1 KD, Western blotting revealed notable alterations among migration-related proteins, including E-cadherin, N-cadherin, and vimentin (Fig. 2N). These results indicate that SND1 plays a critical regulatory role in PCa proliferation and migration in vitro.

**Fig. 3:**
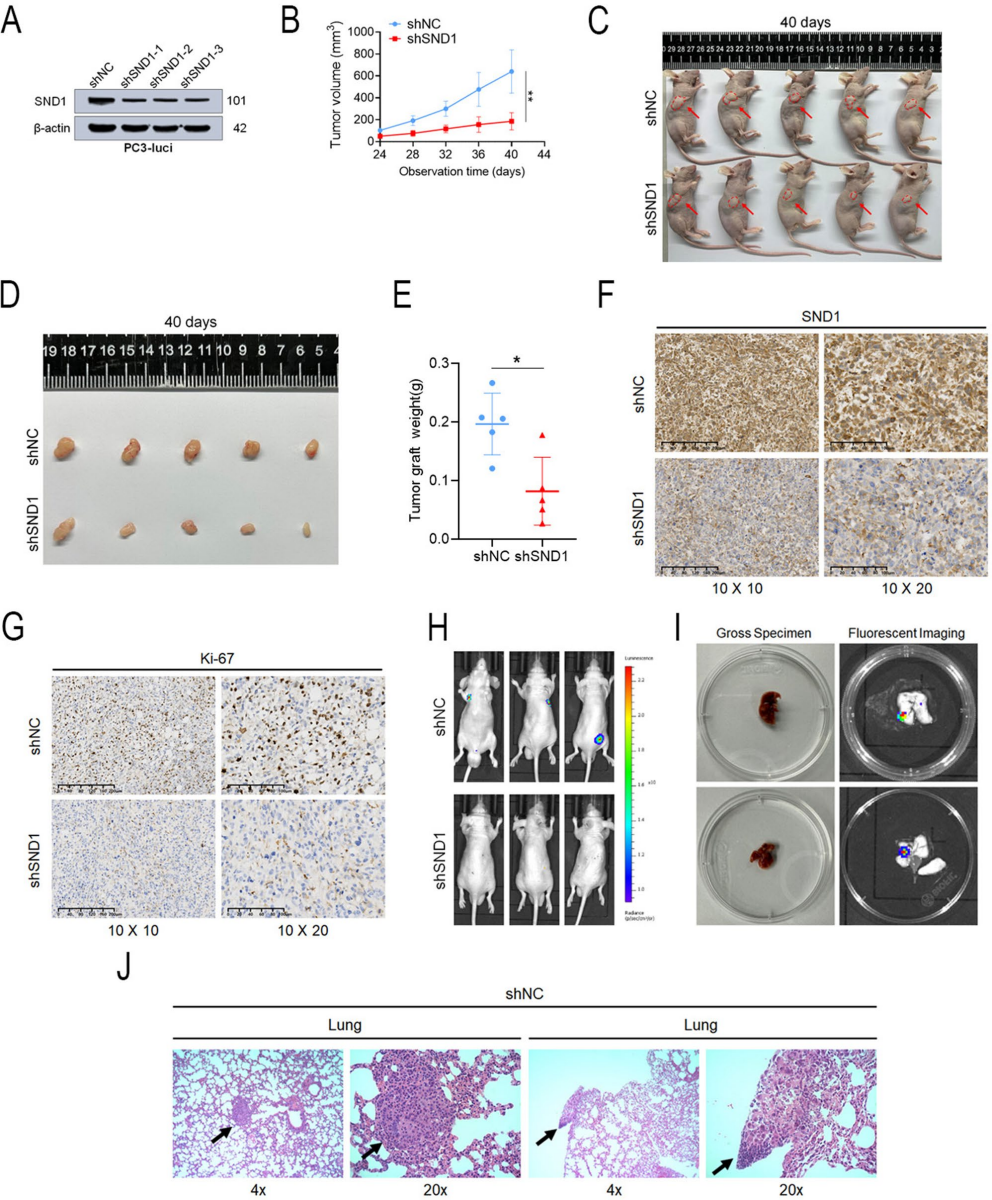
Knockdown of SND1 inhibits tumor growth and metastasis in vivo. **A** SND1 was stably knocked down using lentivirus-based shRNA technique in continuously expressing luciferase PC-3 cell lines (PC3-luci). **B** The growth curve of tumors was plotted (*n* = 5 each group) by measuring the tumor size with electronic vernier caliper every 4 days. **C** Subcutaneous xenografts were photographed and recorded at the end of observation. **D** The nude mice were first anesthetized and then subjected to dissection to obtain subcutaneous tumors, which were measured for size. **E** The dissected tumors were weighed in each group. **F**, **G** IHC staining of SND1 and Ki-67 in tumors were conducted (scale bar = 100 μm). **H** Intravenous tail vein tumor metastasis models in vivo were imaged at 6th weeks. **I** All imaged metastatic organs were anatomized and imaged again to confirm the metastatic sites. **J** H&E staining of metastasis (lung) were made to validate the metastatic tumor tissues. β-actin was the internal reference

Stable SND1 KD PC-3 cells were generated, and KD efficacy was confirmed (Fig. 3A) to explore its biological function in vivo. In subcutaneous transplantation models, stable SND1 KD significantly inhibited tumor growth in vivo (Figs. 3B–E). Western blotting confirmed significant SND1 KD at the protein level in the shSND1 group (Fig. S2H). IHC staining revealed reduced expression of SND1 and Ki-67 in the shSND1 group (Figs. 3F-G). In metastatic models, the shNC group had a higher number of metastatic sites (3/5 versus 0/5) compared to the shSND1 group at six weeks post-injection (Fig. 3H). Imaging of metastatic sites was performed, followed by H&E staining (Figs. 3I-J). In conclusion, SND1 KD suppressed tumor growth and metastasis in vivo.

### SREBF1 activated SND1 transcription in PCa

**Fig. 4:**
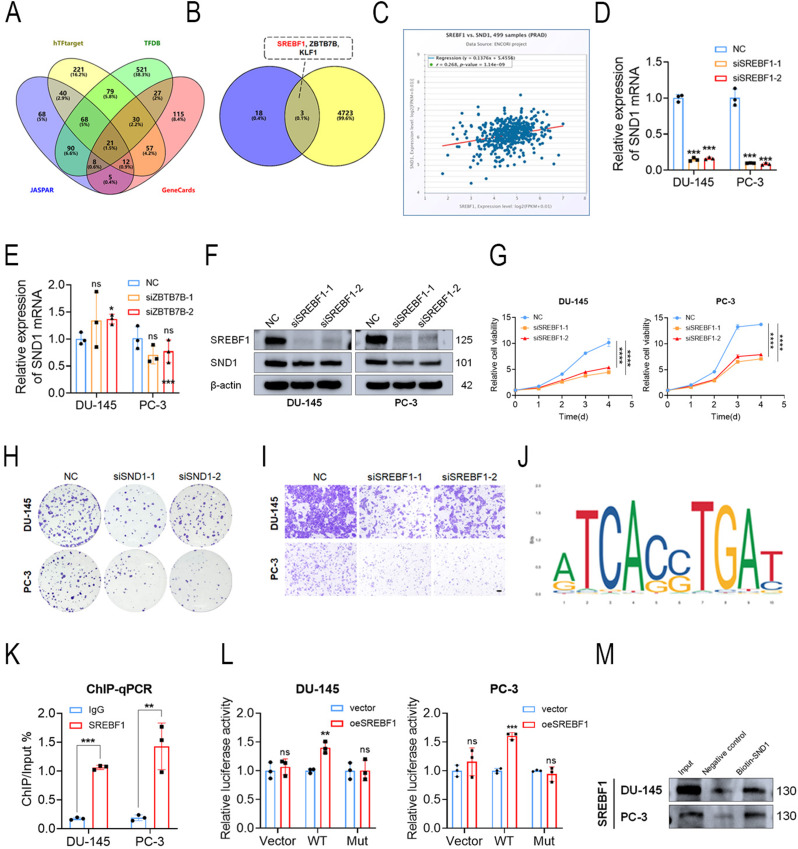
SREBF1 activates the transcription of SND1 in Pca **A** JASPAR, hTFtarget, TFDB and GeneCards were used to predict potential TFs of SND1. **B** Three TFs were screened out according to data from Linkedomics combined with online websites. **C** Correlation analysis was made between SND1 and SREBF1. D, **E** Expression level of SND1 mRNA was evaluated with qRT-PCR after knocking down SREBF1 or ZBTB7B with siRNAs. **F** Expression level of SND1 was evaluated with western blot after knocking down SREBF1 with siRNAs. **G**-**I** Knockdown of SREBF1 suppressed PCa proliferation and migration ability evaluated by CCK-8, colony formation assay and trans-well assay. **J** The motif of binding sites was downloaded from JASPAR. **K** ChIP-qPCR was performed and analyzed using SREBF1 antibody. **L** Dual luciferase reporter assay was conducted driven by SREBF1 plasmid and the SND1 promoters. **M** DNA pull-down assay was performed using biotin-labeled probe. β-actin was the internal reference

Given SND1 upregulation in PCa, we hypothesized that its transcription may be activated by factors such as ELK1 [30]. Using online tools, including JASPAR, hTFtarget, TFDB, and GeneCards, we identified 21 potential transcription factors for SND1 promoters (Fig. 4A). We also analyzed data from the LinkedOmics database to identify genes that are positively correlated with SND1 expression. This led to the identification of three overlapping genes, SREBF1, ZBTB7B, and KLF1 (Fig. 4B), which were also upregulated in PCa according to TCGA data (Figs. S2I–K). Data from ENCORI indicated that SND1 expression was positively correlated with SREBF1, ZBTB7B, and KLF1 in PCa (Figs. 4C and S2L-M). To further identify the transcription factors targeting the SND1 gene, we first excluded KLF1 due to its extremely low expression in PCa and its weak positive correlation with SND1. Next, we assessed SND1 mRNA expression by qRT-PCR following KD of SREBF1 and ZBTB7B using siRNAs. The results indicated that SND1 was suppressed after SREBF1 KD but unaffected by ZBTB7B KD (Figs. 4D-E). Similar results were observed in Western blotting after SREBF1 KD (Fig. 4F). A series of functional assays demonstrated reduced proliferation and migration of PCa cells upon SREBF1 KD (Figs. 4G–I and S2N-O).

To further confirm the regulatory effect of the transcription factor SREBF1 on the SND1 promoter region, we first performed an SREBF1 ChIP-seq, and the results revealed that peaks in the SND1 promoter region were consistent with the binding sequence provided by online websites (Fig. 4J). Next, we conducted ChIP-qPCR and confirmed that SREBF1 directly binds to the SND1 promoter (Fig. 4K). To pinpoint the specific binding region, a dual-luciferase reporter assay was performed, which suggested that SREBF1 overexpression increased the luciferase activity of the wild-type reporter while having no effect on the mutant reporter (Fig. 4L). Additionally, a biotin-labeled SND1 promoter probe was designed to capture SREBF1, and a subsequent DNA pull-down assay validated the interaction between SREBF1 and the SND1 promoter region (Fig. 4M). These findings suggest that SREBF1 functions as an oncogenic transcription factor that activates SND1 transcription.

### SND1 regulated the SESN2 expression level through MTDH interaction

**Fig. 5:**
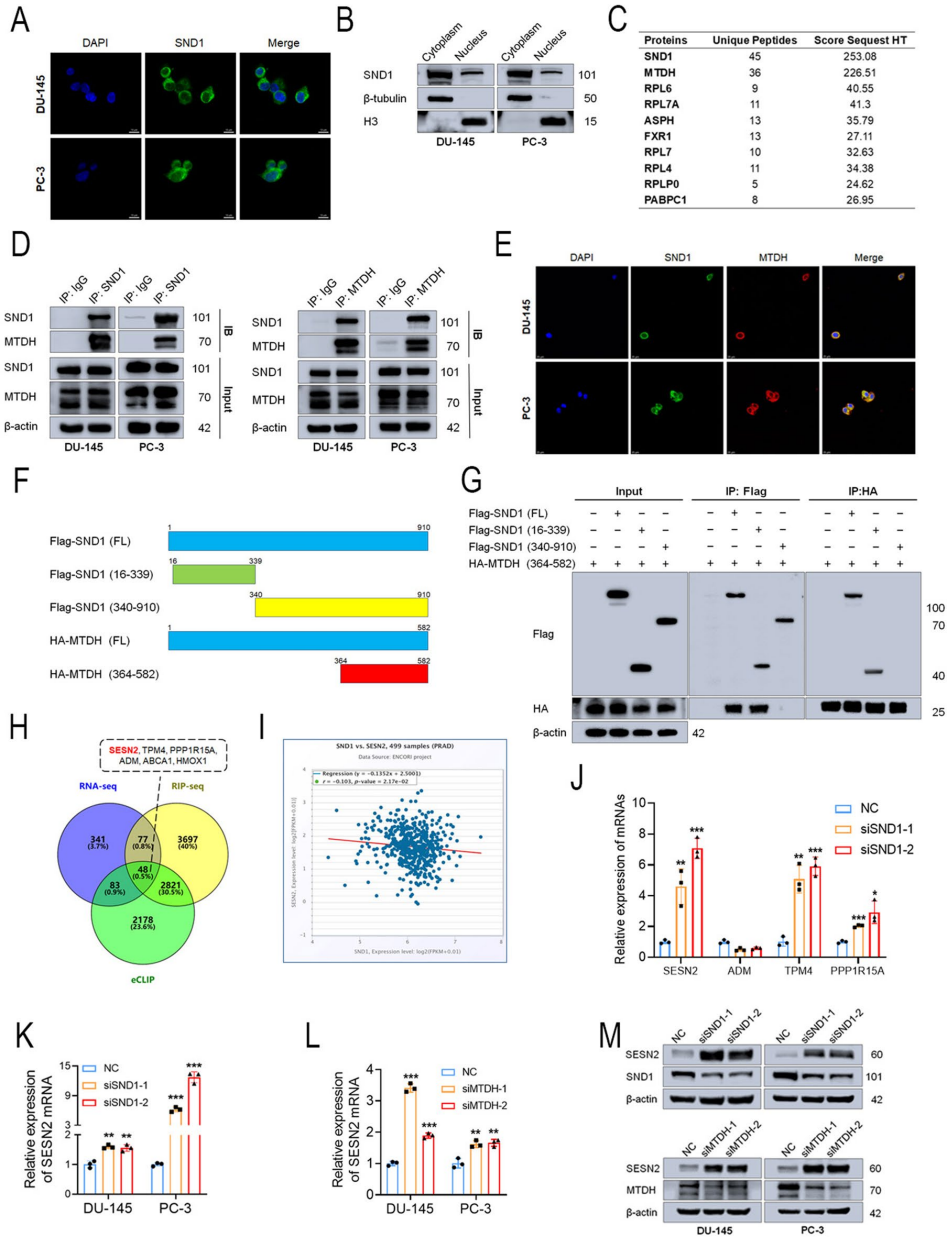
SND1 regulates the expression level of SESN2 through MTDH interaction **A** IF showed the subcellular localization of SND1. **B** Isolation of nuclear and cytoplasmic extract showed the localization of SND1 protein in PCa cells. **C** Proteins with the highest binding abundance to SND1 identified by LC/MS were listed. **D** Co-IP was performed to validate the interaction between MTDH and SND1. **E** IF was performed to validate the interaction between MTDH and SND1. **F** The diagram of truncated plasmids was as presented. **G** Co-IP was performed to validated the region of SND1 binding to MTDH. **H** Venn diagram suggested the number of overlapping genes preliminarily screened by eCLIP-seq, RNA-seq and RIP-seq (48 up). **I** Correlation analysis was made between SND1 and SESN2. **J** The expression of potential target mRNAs was detected by qRT-PCR upon SND1 KD. **K**, **L** The mRNA level of SESN2 was detected by qRT-PCR upon SND1 KD or MTDH KD. **M** The protein level of SESN2 was detected by western blot upon SND1 KD or MTDH KD. β-actin was the internal reference

Previously, most research on SND1 has focused on its transcriptional regulatory functions [31, 32]. Recently, numerous studies have documented that SND1 functions as an RBP to regulate RNA metabolism, such as RNA stability, translation, and splicing [33]. To clarify the distribution of SND1 in PCa cells, we performed IF and nuclear-cytoplasmic protein extraction assays, which revealed that SND1 was predominantly localized in the cytoplasm (Figs. 5A-B). However, the specific mechanisms by which SND1, an RNA-binding protein, exerts its pro-oncogenic effects in PCa remain unclear. To identify proteins that cooperate with SND1 in regulating RNA metabolism, Co-IP-LC/MS analysis was performed, and mass spectrometry identified MTDH as the most abundant interacting protein pulled down by SND1, ranking first among all the bound proteins (Fig. 5C). Based on these findings, we focused our investigation on the interaction between SND1 and MTDH.

Previous studies have reported that MTDH interacts with SND1 to promote breast cancer progression [34]. However, this interaction has not been confirmed in PCa. We conducted Co-IP and IF experiments, which demonstrated that SND1 interacts with MTDH (Figs. 5D-E). Based on the report by Guo et al. [35], we designed full-length SND1 plasmids with a FLAG tag and truncated SND1 plasmids (16–339, 340–910), along with a truncated MTDH plasmid (364–582) carrying an HA tag (Fig. 5F), and Co-IP experiments were performed. Consistent with previous findings, the 16–339 region of SND1 interacted with the 364–582 region of MTDH (Fig. 5G). Further functional assays and Western blotting revealed that MTDH KD inhibited PCa cell proliferation and migration by affecting cell cycle- and migration-related protein expression (Figs. S3A–F). Our results suggest that SND1 interacts with MTDH to promote PCa progression.

To explore the mRNA regulatory role and screen potential downstream targets of SND1, mRNA sequencing and RIP sequencing were conducted in PC-3 cells. Scadden et al. have reported SND1 as a component of the RISC complex, promoting the cleavage of double-stranded RNA [8]. Additionally, an important study conducted by Shen et al. suggests that the MTDH-SND1 complex mediates Tap1/2 mRNA degradation [36]. Therefore, we focused on mRNAs that were upregulated upon SND1 KD. By integrating RIP-seq results with eCLIP data, we identified 48 candidate genes and focused on the top ten genes with the most significant upregulation following SND1 KD (Fig. 5H). We then performed correlation analyses with SND1. The results revealed that SESN2, TPM4, ADM, PPP1R15A, ABCA1, and HMOX1 were significantly negatively correlated with SND1 (Figs. 5I and S3G–K). ABCA1 and HMOX1 were ruled out for upregulation in PCa according to the TCGA database. Subsequent PCR validation revealed increased expression of SESN2, TPM4, and PPP1R15A after SND1 KD, with SESN2 exhibiting the most significant change, while ADM expression remained unchanged (Fig. 5J). Therefore, we preliminarily selected SESN2 as a downstream target regulated by SND1, which has been reported to be downregulated in various cancers and to inhibit bladder cancer progression, but its role in PCa remains unclear [24, 37]. Consequently, we hypothesized that SESN2 may exert a tumor-suppressive role in PCa and that the MTDH-SND1 complex targets SESN2 mRNA for degradation, thereby promoting PCa progression. Further experiments demonstrated that KD of either SND1 or MTDH significantly promoted SESN2 expression, confirming their regulatory effect on SESN2 (Figs. 5K–M).

**Fig. 6:**
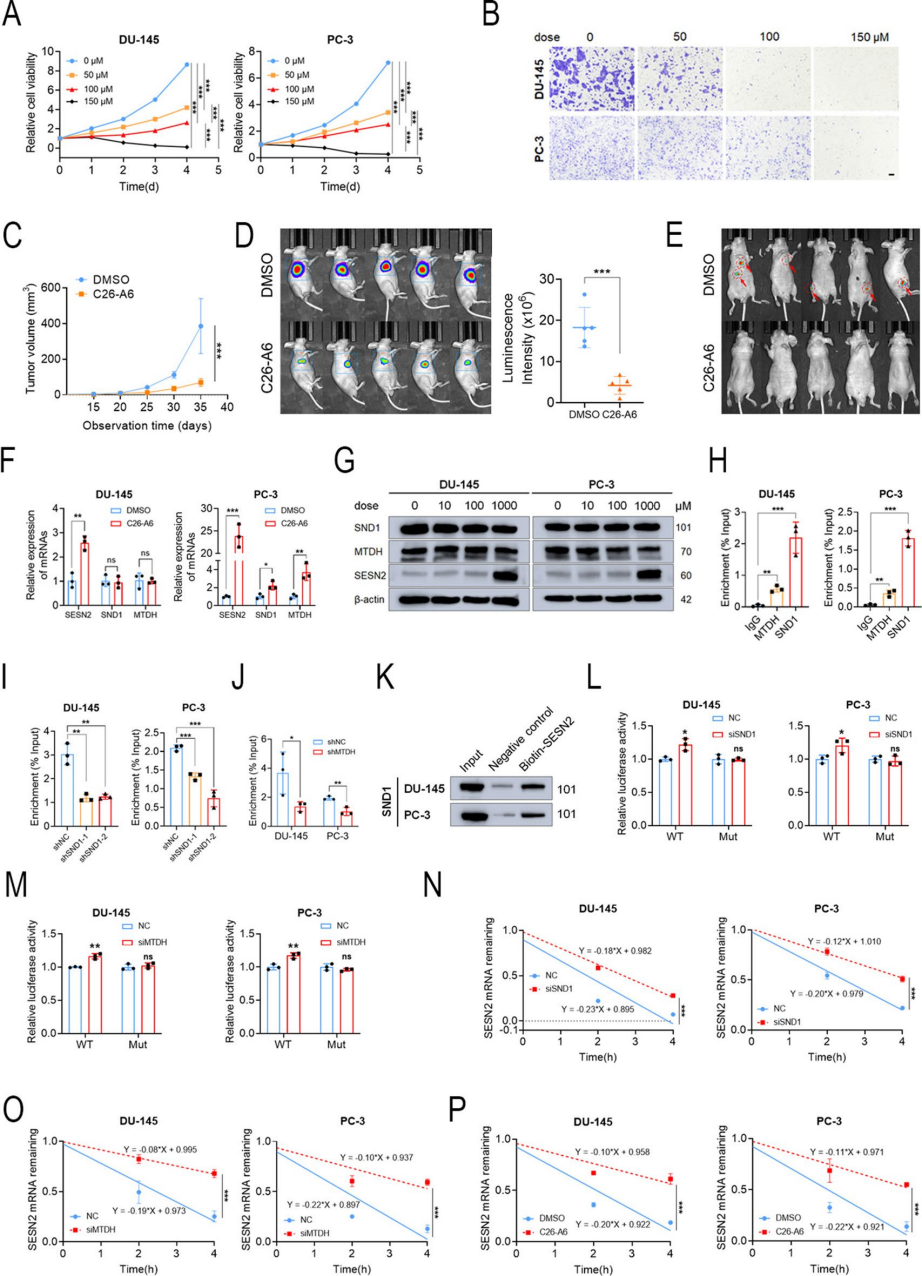
MTDH-SND1 complex promotes the decay of SESN2 mRNA while C26-A6 suppresses this effect **A**, **B** C26-A6 inhibited the proliferation and migration of PCa in vitro. **C**-**E** C26-A6 inhibited the proliferation and migration of PCa in vivo.**F**, **G** C26-A6 significantly enhanced SESN2 mRNA and protein levels. **H** RIP-PCR assay clarified the association of MTDH, SND1 and SESN2 mRNA. **I** RIP-PCR assay clarified the regulation of MTDH to SESN2 depended on SND1. **J** RIP-PCR assay clarified the regulation of SND1 to SESN2 depended on MTDH. **K** RNA pull-down assay confirmed the interaction between SND1 and SESN2 mRNA. **L** Relative luciferase activities of SESN2-WT and SESN2-Mut in SND1 KD or MTDH KD cells were detected. **M**-**P** RNA stability assays revealed that SND1 KD, or MTDH KD, or C26-A6 prolonged SESN2 mRNA half-life. β-actin was the internal reference

Meng et al. employed a small-molecule screening system to identify C26-A6 as a specific inhibitor that disrupts MTDH-SND1 protein-protein interactions. It has been reported that C26-A6 significantly inhibited triple-negative breast cancer growth and metastasis. However, its pharmacological effects on the MTDH-SND1 complex in PCa remain unexplored. We first determined the half-maximal inhibitory concentration values of C26-A6 in DU-145 and PC-3 (Fig. S4A). Subsequently, functional experiments demonstrated that the proliferation and migration abilities were gradually inhibited with increasing drug concentrations (Figs. 6A-B and S4B-D). Similarly, C26-A6 suppressed tumor growth and metastasis in vivo (Figs. 6C-E and S4E-G). Furthermore, we found that disrupting the MTDH-SND1 protein interaction with C26-A6 enhanced SESN2 mRNA and protein levels (Figs. 6F-G). Our preliminary findings suggest that disrupting the MTDH-SND1 complex with C26-A6 also modulates SESN2 expression and influences PCa progression.

### MTDH-SND1 complex facilitated the SESN2 mRNA degradation

Shen et al. reported that the MTDH-SND1 complex directly interacts with and degrades Tap1/2 mRNA, thereby impairing tumor antigen presentation and suppressing T-cell activation. To explore the role of the MTDH-SND1 complex on SESN2 mRNA in PCa, we first conducted RIP-qPCR to assess the interaction between SND1 and SESN2 and between MTDH and SESN2. The results revealed that SESN2 mRNA was significantly more enriched by SND1 or MTDH compared to IgG (Fig. 6H). Using lentivirus-mediated KD of SND1 or MTDH, we observed that SND1 KD significantly inhibited the binding of MTDH to SESN2 mRNA, suggesting that SND1 is essential for the association between MTDH and SESN2. Conversely, in tumor cells with MTDH KD, the binding of SND1 to SESN2 was notably weakened, indicating the crucial role of MTDH in promoting the SND1-SESN2 interaction (Figs. 6I-J). RNA pull-down assay was also performed to examine the binding in the reverse direction, which exhibited that biotin-labeled SESN2 RNA oligos could bind to SND1 (Fig. 6K).

Additionally, subsequent dual-luciferase reporter assays revealed that inhibiting SND1 or MTDH significantly amplified the luciferase activity of SESN2 wild-type reporters but did not affect that of SESN2 mutant reporters (Figs. 6L-M and S4H). We further treated cells with actinomycin D at 4, 2, and 0 h before collection, and subsequent PCR results revealed that KD of SND1 or MTDH or treatment with C26-A6 inhibited the decay rate of SESN2 mRNA (Figs. 6N-P). These results suggest that MTDH and SND1 can directly bind to SESN2 mRNA and that the negative regulation of SESN2 is dependent on the formation of the MTDH-SND1 complex.

### SESN2 was responsible for SND1 and MTDH-induced regulation of PCa progression

**Fig. 7:**
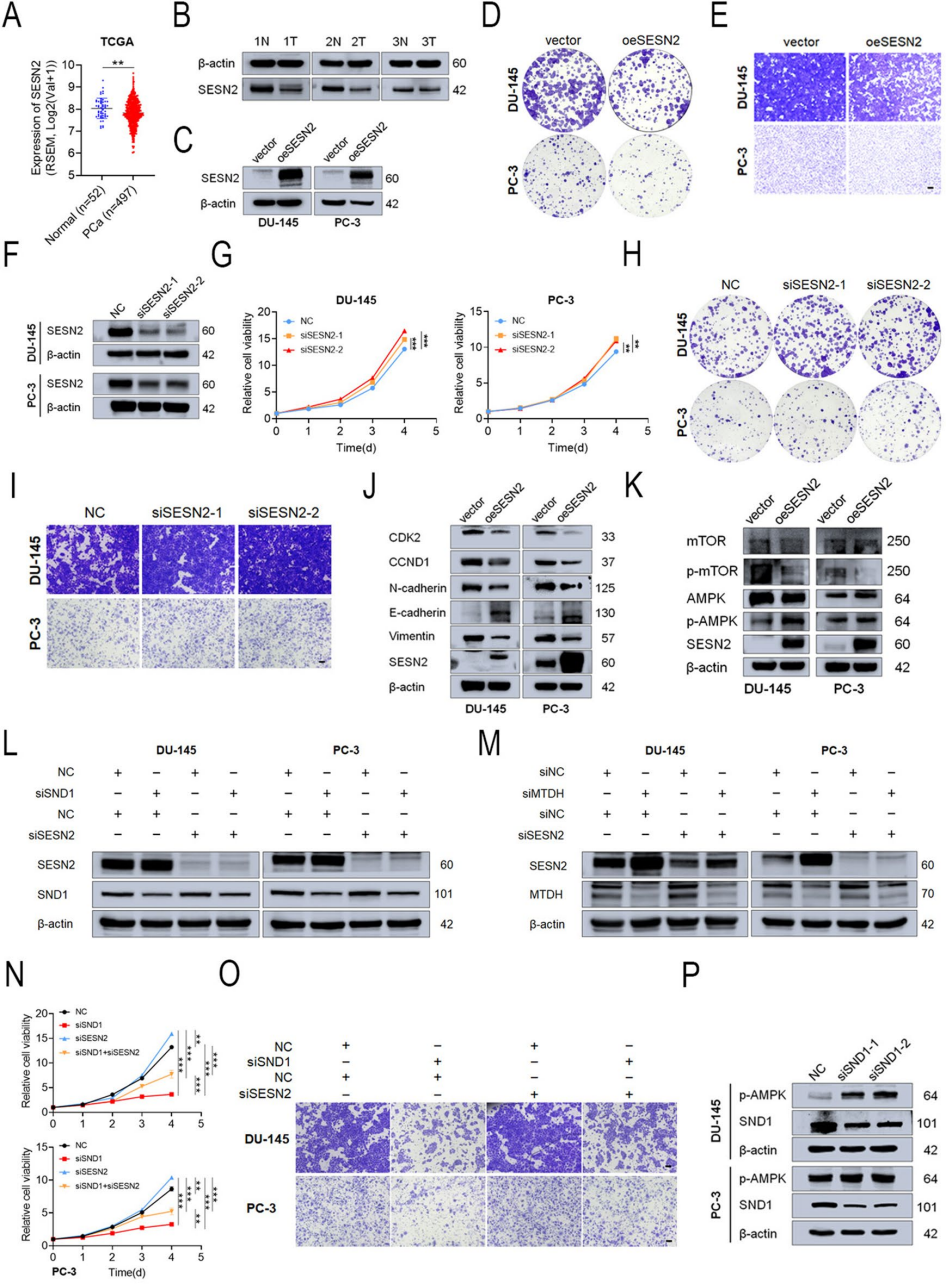
SESN2 are responsible for SND1 and MTDH induced regulation of PCa progression **A** The expression pattern of SESN2 was plotted in 497 PCa tissues and 52 normal prostate tissues using data downloaded from TCGA database. **B** Three pairs of PCa samples were subject to western blot analysis of SND1. **C**-**E** Overexpression of SESN2 inhibited PCa tumor growth and metastasis accessed by colony formation assay and trans-well assay (scale bar = 250 μm). **F**-**I** Knockdown of SESN2 promoted PCa tumor growth and metastasis accessed by CCK-8, colony formation assay and trans-well assay (scale bar = 250 μm). **J** The expression of CDK2, CCND1, E-cadherin, N-cadherin and vimentin was detected by western blot after overexpressing SESN2. **K** The expression of AMPK, p-AMPK, mTOR and p-mTOR were detected by western blot after overexpressing SESN2. **L**, **M** Western blot showed knockdown of SESN2 partly reversed the increase of SESN2 expression induced by SND1 KD or MTDH KD. **N**, **O** CCK-8 and trans-well assay after SND1 knockdown alone or co-transfected with siSESN2 (scale bar = 250 μm). **P** The expression of p-AMPK after knocking down SND1 was detected. β-actin was the internal reference

Several studies confirmed that SESN2 promoted apoptosis of various cancer cell types, including lung adenocarcinoma, colon cancer, human head and neck cancer, and others [37, 38, 39]. However, the role of SESN2 in PCa remained elusive. Analysis of the TCGA database revealed that SESN2 is downregulated in PCa tissues compared to benign prostate tissues (Fig. 7A), which was also confirmed in clinical samples from our center (Fig. 7B). A series of functional experiments exhibited that SESN2 overexpression inhibited PCa progression (Figs. 7C-E and S5A-B) while silencing SESN2 yielded the opposite results (Figs. 7F-I and S5C-D). Furthermore, SESN2 overexpression suppressed the protein level of cell cycle-related proteins and mesenchymal markers, thereby suppressing cell cycle and epithelial-mesenchymal transition (Fig. 7J).

The SESN2/AMPK/mTOR pathway is essential for regulating cellular energy metabolism, stress response, and cancer progression [26]. Yu et al. reported that miR-615-3p promotes osteosarcoma progression by inhibiting this signaling pathway [40]. Consequently, we examined the expression and activation of proteins in this pathway. The results revealed that SESN2 overexpression led to the phosphorylation of AMPK at Thr172, activating AMPK and partially inhibiting mTOR signaling (Fig. 7K). Collectively, these findings suggest that SESN2 plays a suppressive role in PCa progression by activating the AMPK/mTOR pathway.

To further clarify whether the promotion of PCa progression by SND1 and MTDH depends on SESN2, rescue experiments were conducted. CCK-8 and colony formation assays indicated that KD of SND1 or MTDH greatly inhibited PCa cell proliferation, which was partially rescued by SESN2 KD (Figs. 7N and S5E-G). Similar results were observed in the Transwell assays (Figs. 7O and S5H). The results of the functional assays were confirmed by Western blotting (Figs. 7L-M). Finally, after SND1 KD, we observed phosphorylation and activation of AMPK at Thr172, further confirming our hypothesis (Fig. 7P).

Our study suggests that in PCa, the SND1 gene is transcriptionally activated by SREBF1, which interacts with MTDH and degrades SESN2 mRNA, thereby inhibiting AMPK activation and promoting mTOR signaling. This signaling pathway can be reversed by the MTDH-SND1-specific inhibitor C26-A6. The proposed SREBF1/SND1/SESN2/AMPK/mTOR pathway may provide new insights into PCa treatment.

## Discussion

To date, the oncogenic role of SND1 in various cancers is relatively well established; however, the underlying mechanism remains primarily focused on transcriptional regulation. Wu et al. reported that the SND1-KDM6A complex recruits RPA and Ku70 to nascent DNA strands, preventing replication fork collapse, enhancing genomic stability, and promoting chemoresistance in esophageal squamous cell carcinoma [41]. Liao et al. reported that the oncoprotein ERG binds to the MTDH-SND1 complex through the Tudor domain of SND1, thereby promoting the nuclear localization of SND1/MTDH and significantly enhancing PCa growth [32]. Jariwala et al. reported that SND1, as a subunit of RISC, promotes hepatocellular carcinoma progression by activating the AKT and NF-κB signaling pathways, and the selective SND1 inhibitor 3’,5’-deoxythymidine bisphosphate (pdTp) can effectively suppress this effect [42]. Yu et al. reported that SND1 directly binds to the histone acetyltransferase GCN5 and recruits it to the promoter regions of Smad2/3/4, thereby enhancing the transcriptional activation of Smad2/3/4 genes, potentiating the TGFβ1 signaling pathway, and promoting breast cancer metastasis [43]. These studies indicate that SND1 is involved in the occurrence and development of multiple tumors, including prostate, liver, esophageal, and breast cancers. The underlying mechanisms include transcriptional regulation and modulation of genomic stability, among others.

Additionally, SND1 has been reported to participate in regulating RNA alternative splicing, maturation, and stability, thereby promoting tumor progression. Zhou et al. reported that after the interaction between SND1 and MTDH was weakened, the activity of RISC was significantly reduced, and the expression of tumor suppressor genes such as PTEN and CDKN1A was upregulated, thereby inhibiting the progression of hepatocellular carcinoma [44]. Cappellari’s research indicated that SND1 regulates the alternative splicing of CD44 pre-mRNA through SAM68, promoting the inclusion of CD44 variable exons, which facilitates the progression of PCa. Zhang et al. reported that SND1 binds to and stabilizes YWHAB mRNA, while circSMARCA5 inhibits the binding of SND1 to YWHAB, suppresses the proliferation and invasion, and promotes apoptosis of cervical cancer cells, thereby inhibiting the metastasis of cervical cancer [45]. Importantly, Wan et al. reported MTDH-SND1 complex is essential for the expansion and activity of tumor-initiating cells in various oncogene- and carcinogen-induced mammary tumors [34].

Moreover, pharmacological disruption of the MTDH-SND1 complex enhances tumor antigen presentation and synergizes with anti-PD-1 therapy in metastatic breast cancer [36]. These studies on SND1 have shifted the focus from transcriptional regulation and genomic stability to the interaction between SND1 and RNA, including RNA stability, alternative splicing, m6A modification, and others. Among these, the interaction between SND1 and MTDH is particularly important, as it plays a crucial role in SND1’s involvement in regulating RNA metabolism.

To date, studies on SND1 exerting transcriptional regulatory functions to promote PCa progression have been relatively well established [32, 46]. However, the precise molecular mechanisms through which SND1, as an RBP, facilitates PCa progression remain unclear. Our study demonstrates that in DU-145 and PC-3 cells, SND1 is predominantly localized in the cytoplasm, offering new insights into the molecular mechanisms by which SND1 promotes oncogenesis in PCa.

Although extensive research has been conducted on the expression patterns and biological functions of SND1 in various cancers, the underlying mechanisms driving overexpression remain unclear. It has been reported that Smads and c-Myb function as regulatory factors located upstream of the distal region of the SND1 promoter [47, 48]. However, Navarro-Imaz et al. characterized the proximal SND1 promoter [49], which facilitates the binding of the basal transcription factors nuclear factor-Y and specificity protein 1 [50, 51]. Our study provides preliminary evidence suggesting that SREBF1 is vital for the transcriptional activation of SND1.

Our study focused on the regulatory role of SND1 in RNA functions, particularly RNA stability. As a selective inhibitor of SND1, 3’,5’-deoxythymidine bisphosphate (pdTp) can inhibit the function of SND1 as a subunit of the RISC complex, thereby exhibiting antitumor activity [52]. This provides a novel approach for inhibiting SND1-mediated tumorigenesis. Furthermore, SND1 often collaborates with other proteins to regulate RNA, with MTDH being one of the most crucial partners, as demonstrated by mass spectrometry and numerous studies. Guo et al. determined the high-resolution crystal structure of the MTDH-SND1 complex, revealing the spatial characteristics of their interactions. This provides a solid structural foundation for further investigation into the MTDH-SND1-mediated signaling mechanism and the design of drugs targeting this complex [35]. On this basis, we designed truncated plasmids and conducted Co-IP experiments, confirming the domains involved in the interaction between MTDH and SND1. Moreover, Shen et al. identified C26-A2 and C26-A6 as specific inhibitors of the MTDH-SND1 complex using a small-molecule compound screening system, which significantly suppressed TNBC growth and metastasis [16]. Further studies revealed that the MTDH-SND1 complex binds to Tap1/2 mRNA and promotes its degradation, thereby reducing tumor antigen presentation and inhibiting T-cell infiltration and activation [36]. These findings suggest that targeting the MTDH-SND1 complex with specific inhibitors may be a promising therapeutic strategy for TNBC and metastatic breast cancer. However, the efficacy of these inhibitors in PCa, particularly in CRPC, remains unclear. Our findings offer preliminary insights into the understudied aspects of using C26-A6 for PCa treatment.

The evolutionarily conserved stress-induced protein SESN2 belongs to the sestrin family, and it has been reported to be upregulated in response to various stressors, including DNA damage and hypoxia. Recent studies have provided growing evidence that SESN2 plays a pivotal role in tumor progression. Maiuri et al. reported that SESN2 upregulates autophagy by inhibiting the mTOR signaling pathway [53]. Kim et al. confirmed that oxidative stress-induced SESN2 overexpression inhibits colon cancer growth [54]. Liang et al. also reported that isorhapontigenin-induced activation of SESN2 suppresses bladder cancer progression by activating autophagy [24]. However, the specific role of SESN2 in PCa progression remains unclear. Our preliminary findings suggest that SESN2 exerts anti-cancer effects in PCa, possibly by inhibiting AMPK phosphorylation, leading to the activation of the mTOR signaling pathway.

Above all, our study revealed the upregulation of SND1 in PCa, which is transcriptionally activated by SREBF1. Mechanistically, SND1 interacts with MTDH and promotes SEN2 mRNA degradation, modulating PCa progression through the AMPK/mTOR pathway. Disruption of this complex using C26-A6 exhibited significant antitumor effects in vitro and in vivo. This study has several limitations. First, the analysis of TCGA data revealed a non-significant association between elevated SND1 expression and poorer clinical outcomes in patients with PCa. Furthermore, SND1 expression levels were not correlated with ISUP grade groupings. These findings substantially constrain the potential prognostic utility of SND1 in this malignancy. Second, our sample size was relatively small, which may limit the generalizability of our results. Moreover, incomplete IHC profiling in the PCa cohort restricted molecular subtyping analysis. Finally, our experimental model may not fully recapitulate the complexity of humans.

To date, substantial research progress has been made, and numerous important articles have been published on the role of the MTDH-SND1 complex in breast cancer progression, the design of inhibitors targeting this complex, and their observed tumor-suppressive effects. These findings demonstrate the broad application prospects of drug design targeting this complex. PCa and breast cancer share some common biological behaviors; however, the effects of this drug on PCa have not been fully validated. Our study reveals that inhibiting the formation of the MTDH-SND1 complex with C26-A6 can effectively suppress the growth and metastasis of PCa cells in vitro and in vivo. This suggests the potential for further clinical applications of C26-A6. In subsequent research, we need to collect more clinical samples, improve the pathological results of PCa, and explore the clinical value of C26-A6 in PCa treatment.

## Electronic supplementary material

Below is the link to the electronic supplementary material.

Supplementary Material 1: Figures

Supplementary Material 2: Table

## Data Availability

All statistics of this study are available from the corresponding author upon reasonable request.

## Abbreviations

SND1: Staphylococcal Nuclease and Tudor Domain Containing 1
RBP: RNA Binding Protein
TCGA: The Cancer Genome Atlas
SREBF1: Sterol Regulatory Element Binding Transcription Factor 1
CRPC: Castration-Resistant Prostate Cancer
miRNA: MicroRNA
MTDH: Metadherin
SESN2: Sestrin 2
KD: Knockdown
siRNA: Small interfering RNAs
shRNA: Short hairpin RNAs
CCK-8: Cell Counting Kit-8
ChIP: Chromatin Immunoprecipitation
RIP: RNA Immunoprecipitation
Co-IP: Co-Immunoprecipitation
IF: Immunofluorescence
TNBC: Triple-Negative Breast Cancer
