# Supplementary material for: SREBF1-mediated SND1 transcriptional activation promotes prostate cancer progression via MTDH interaction through the SESN2/AMPK/mTOR axis
Source: J Transl Med. 2025 Aug 7;23:885. doi: 10.1186/s12967-025-06762-2 (PMC12333249; doi:10.1186/s12967-025-06762-2)
Supplement: Supplementary file 1 — Supplementary Material 1: Figures [file 12967_2025_6762_MOESM1_ESM.docx]

**Supplementary Figures**

**Supplementary Figure 1**


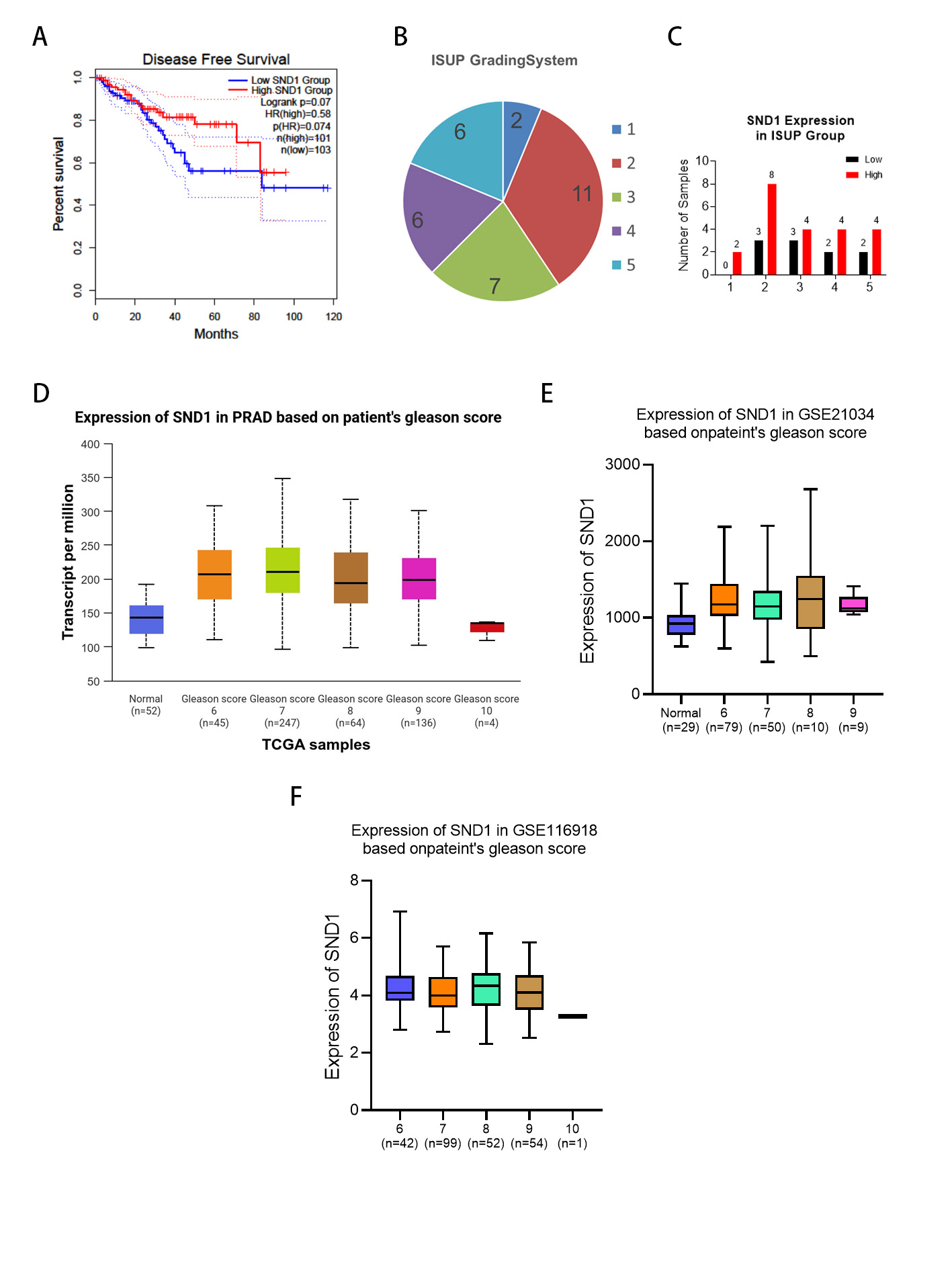


**A** Kaplan-Meier survival analysis of the PCa patients from the TCGA databases classified by SND1 expression (p = 0.07). **B** The ISUP grading system of 32 PCa samples in our center. **C** The expression of SND1 in different ISUP groups. **D-F** The expression pattern of SND1 in separate Gleason score were analyzed by TCGA, GSE21034, GSE116918.

**Supplementary Figure 2**


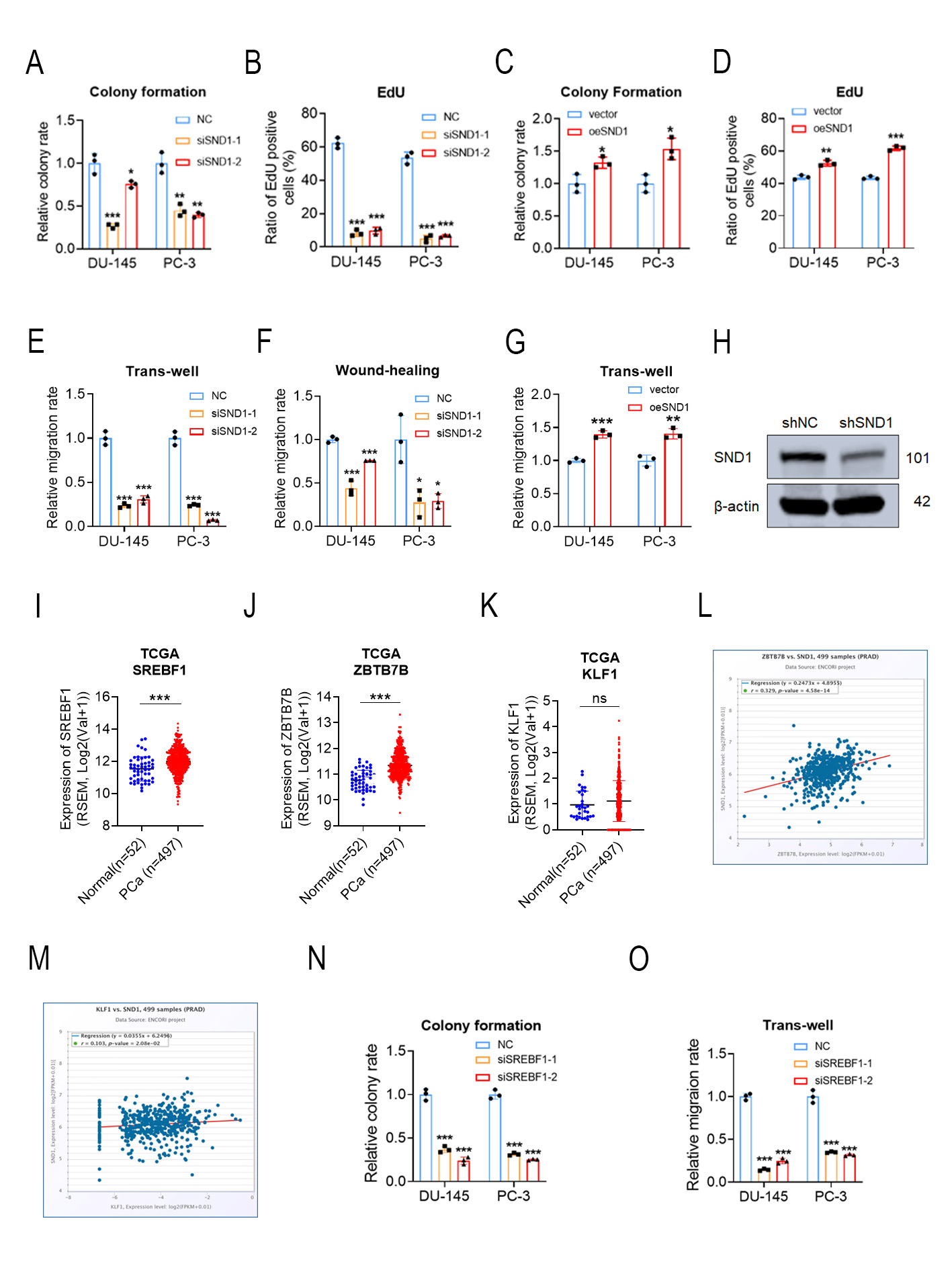


**A** The quantitative statistical results of clone formation assay upon SND1 KD. **B** The quantitative statistical results of EdU assay upon SND1 KD. **C** The quantitative statistical results of clone formation assay after overexpressing SND1. **D** The quantitative statistical results of EdU assay after overexpressing SND1. **E** The quantitative statistical results of transwell assay upon SND1 KD. **F** The quantitative statistical results of wound-healing assay upon SND1 KD. **G** The quantitative statistical results of transwell assay after overexpressing SND1. **H** Protein expression levels of SND1 in subcutaneous xenograft samples were determined using western blot analysis. **I-K** The expression pattern of SREBF1, ZBTB7B, KLF1 was analyzed between 497 PCa tissues and 52 adjacent normal prostate tissues (TCGA database). **L, M** Correlation analysis was made between SND1 and ZBTB7B or KLF1. **N** The quantitative statistical results of clone formation assay upon SND1 KD. **O** The quantitative statistical results of transwell assay upon SND1 KD. β-actin was the internal reference.

**Supplementary Figure 3**


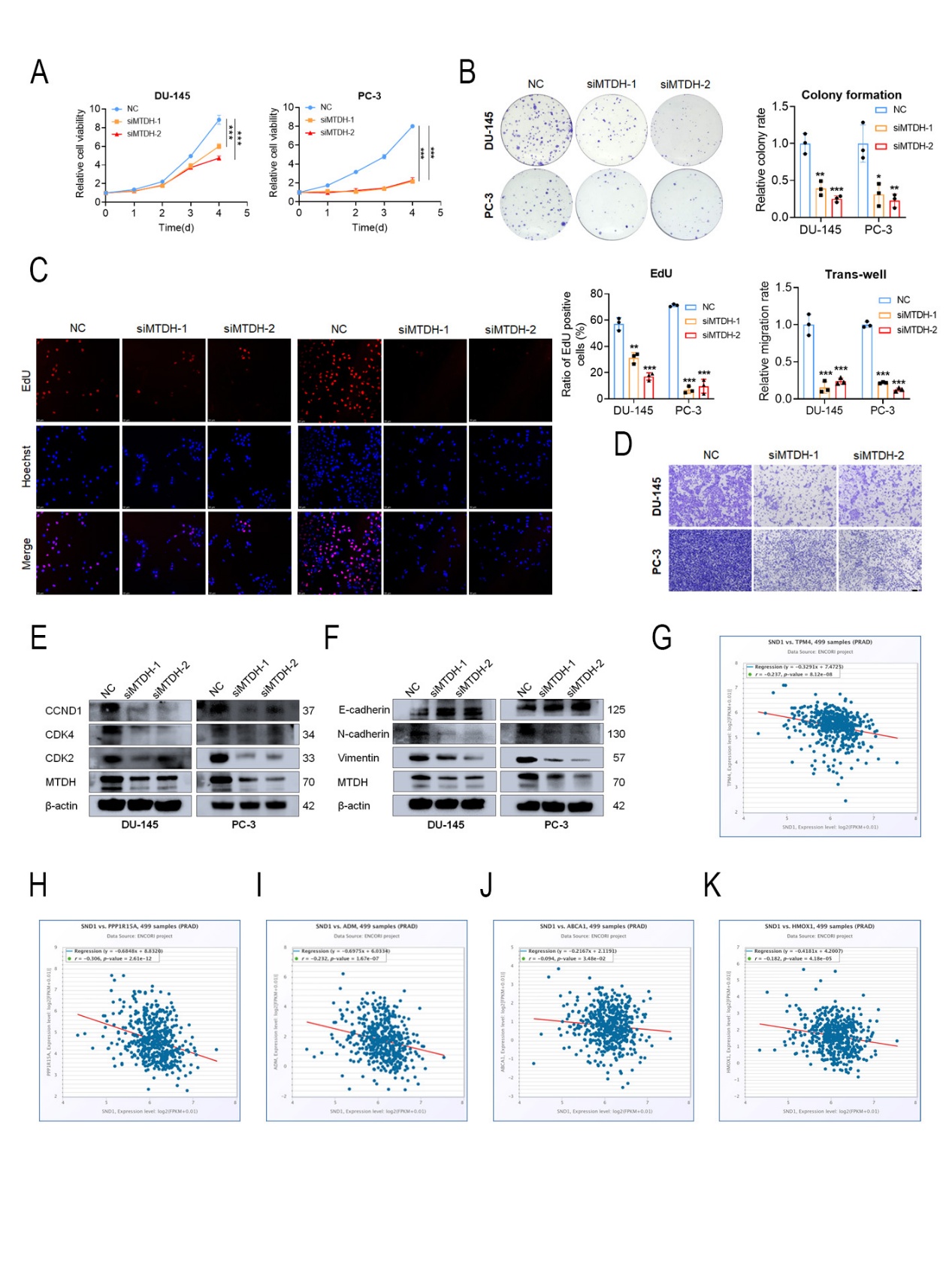


**A-D** Knockdown of MTDH suppressed PCa proliferation and migration evaluated by CCK-8, colony formation, EdU and transwell assay (scale bar = 250 μm). **E** The expression of CDK2, CDK4 and CCND1 were detected by western blot upon SND1 KD. **F** The expression of E-cadherin, N-cadherin and vimentin were detected by western blot upon SND1 KD. **G-K** Correlation analysis was made between SND1 and potential downstream targets such as TPM4, PPP1R15A, ADM, ABCA1, HMOX1. β-actin was the internal reference.

**Supplementary Figure 4**

**
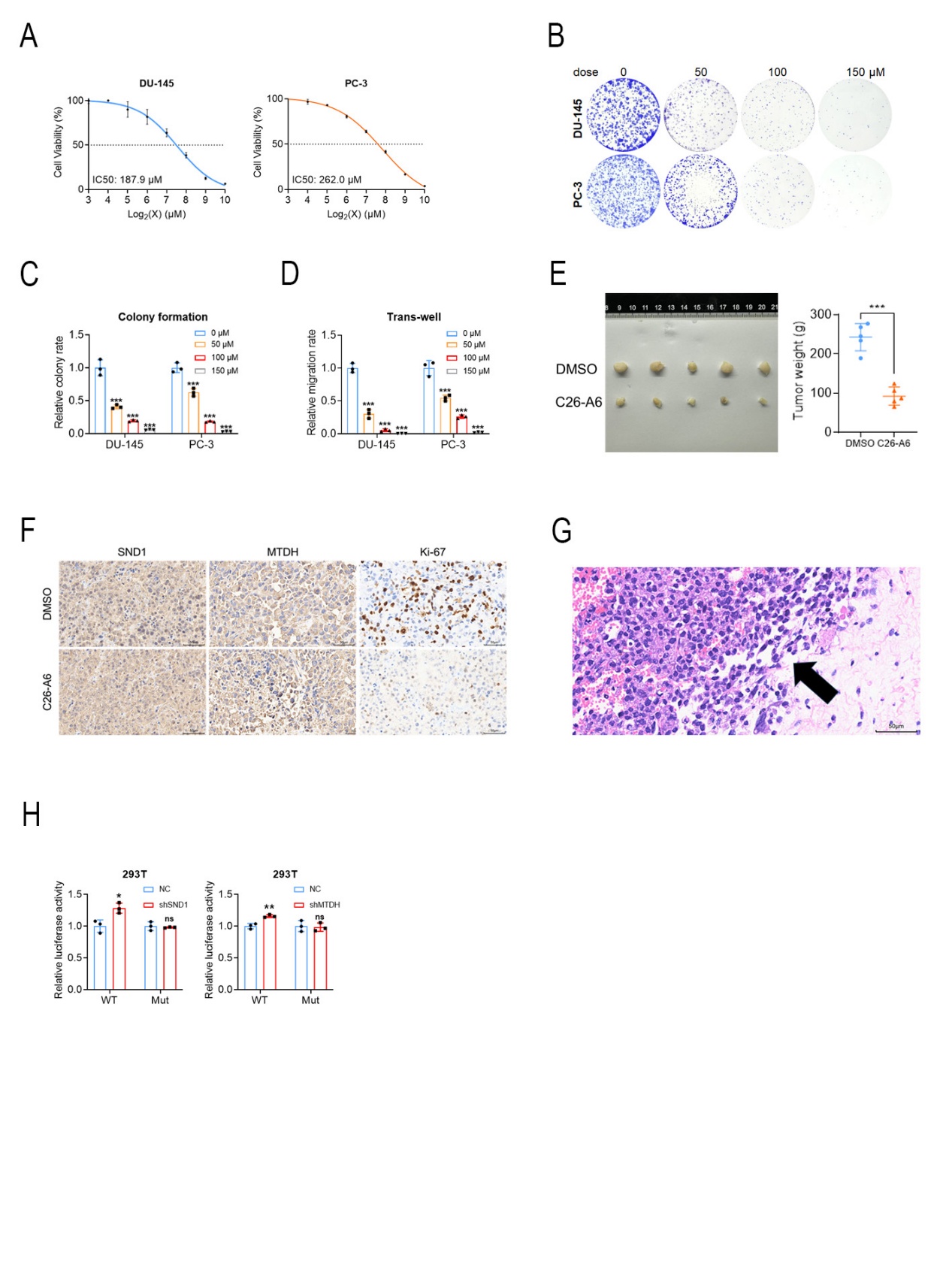
**

**A** IC50 of C26-A6 in DU-145 and PC-3 was detected by CCK-8. **B, C** C26-A6 inhibited the proliferation of PCa verified by colony formation assay. **D** The quantitative statistical results of transwell assay treated with C26-A6 (scale bar = 250 μm). **E** Tumors were weighed after C26-A6 treatment. **F** IHC staining of SND1, MTDH and Ki-67 in tumors were conducted (scale bar = 50 μm). **G** H&E staining of metastasis were made to validate the metastatic tumor tissues. **H** Relative luciferase activities of SESN2-WT and SESN2-Mut were detected in 293T cells.

**Supplementary Figure 5**


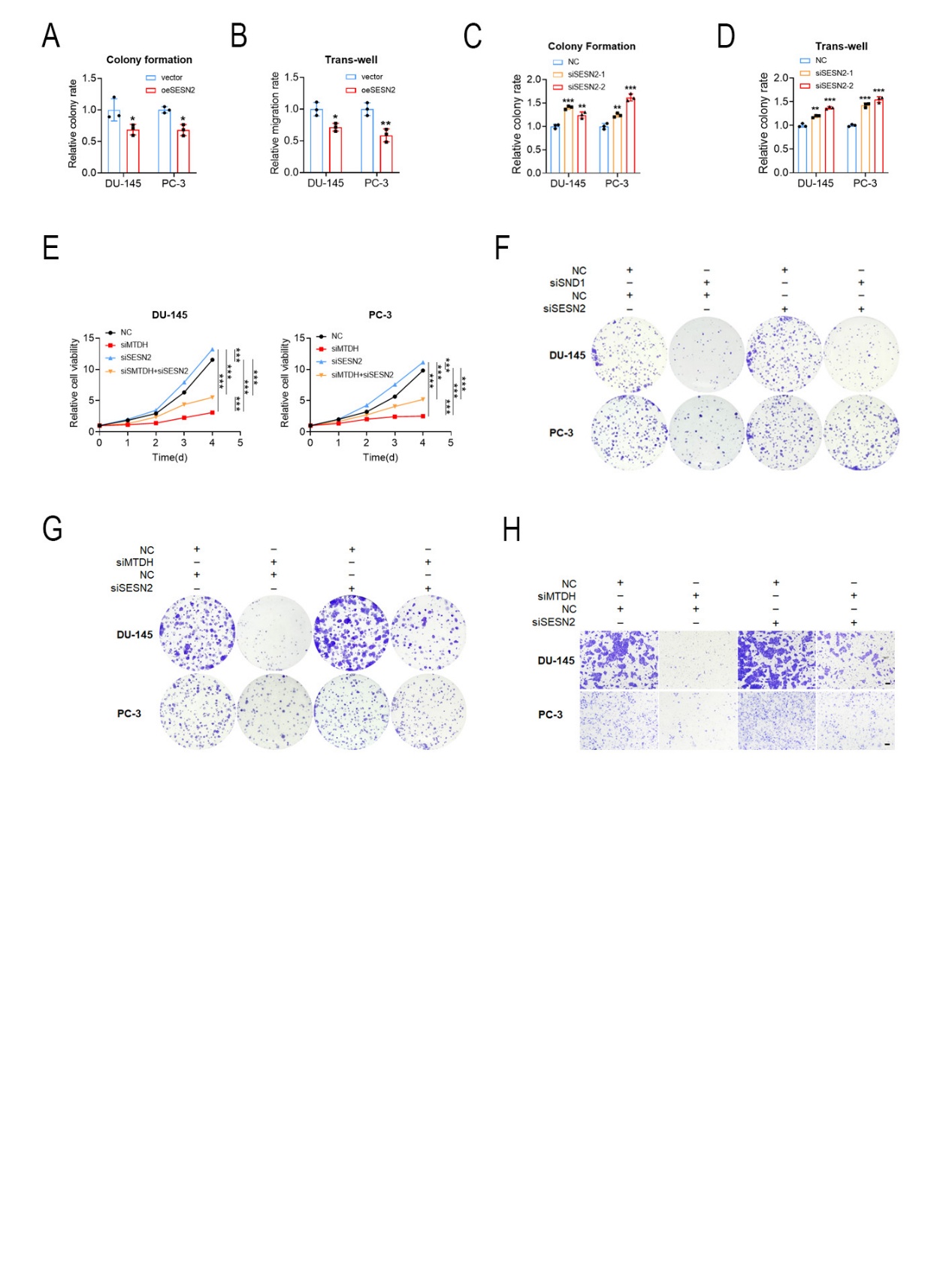


**A, B** The quantitative statistical results of clone formation or transwell assay upon overexpressing SESN2. **C, D** The quantitative statistical results of clone formation and transwell assay upon SESN2 KD. **E** CCK-8 assay after MTDH KD alone or co-transfected with siSESN2. **F, G** Colony formation assay after SND1 KD or MTDH KD alone or co-transfected with siSESN2. **H** Transwell assay after MTDH KD alone or co-transfected with siSESN2 (scale bar = 250 μm).
